# Myc rearrangement redefines the stratification of high‐risk multiple myeloma

**DOI:** 10.1002/cam4.7194

**Published:** 2024-06-07

**Authors:** Xianghong Jin, Hui Li, Dingding Zhang, Shuangjiao Liu, Yuhang Song, Fujing Zhang, Ziping Li, Junling Zhuang

**Affiliations:** ^1^ Department of Hematology, Peking Union Medical College Hospital Chinese Academy of Medical Sciences Beijing China; ^2^ Department of Rare Diseases Peking Union Medical College Hospital, Chinese Academy of Medical Sciences Beijing China; ^3^ Medical Research Center, State Key laboratory of Complex Severe and Rare Diseases, Peking Union Medical College Hospital Chinese Academy of Medical Sciences and Peking Union Medical College Beijing China

**Keywords:** double‐hit, multiple myeloma, *Myc* rearrangement, risk stratification, survival

## Abstract

**Background:**

Myc rearrangement (Myc‐R) is a controversial factor linked to adverse outcomes in newly diagnosed multiple myeloma (NDMM).

**Aims:**

This study aimed to evaluate the impact of Myc‐R on the prognosis of NDMM patients and its role in risk stratification compared with traditional high‐risk cytogenetic abnormalities (HRCAs).

**Materials & Methods:**

A total of 417 NDMM patients enrolled from May 2009 to September 2022 were included. Fluorescence in situ hybridization (FISH) was used to detect Myc‐R and other Myc abnormalities (Myc‐OA). Median progression‐free survival (PFS) and overall survival (OS) were analyzed using Kaplan–Meier methods and log‐rank tests. Multivariate Cox regression analysis was used to identify independent risk factors.

**Results:**

Myc‐R was identified in 13.7% of patients, while 14.6% had Myc‐OA. Patients with Myc‐R had significantly shorter median PFS (15.9 months) and OS (25.1 months) compared with those with Myc‐OA (24.5 months PFS; 29.8 months OS) and Myc‐negative (Myc‐N) status (29.8 months PFS, 29.8 months OS). Myc‐R was independently associated with worse PFS and OS compared to Myc‐OA. Patients with Myc‐R alone had inferior median PFS (15.9 months vs. 28.1 months, *p* = 0.032) and OS (25.1 months vs. 61.2 months, *p* = 0.04) compared to those with traditional single HRCA.

**Discussion:**

The study suggests that traditional single HRCA may not significantly impact survival in NDMM patients. However, incorporating Myc rearrangement or traditional double/triple‐hit HRCAs into the risk stratification model improves its predictive value, highlighting the importance of Myc rearrangement in risk assessment.

**Conclusion:**

Myc rearrangement is an independent adverse prognostic factor in NDMM. The incorporation of Myc rearrangement or multiple HRCAs into risk stratification models improves their prognostic value, providing a novel perspective on high‐risk factors in NDMM.

## 1 INTRODUCTION

Multiple myeloma (MM) is one of incurable plasma cell proliferative disorders, accounting for 1%–2% of all cancers and approximately 10%–18% of all hematologic malignancies.^1^, ^2^ The prognosis of MM patients is highly heterogeneous, ranging from a few months to over a decade.^2^ Currently, several risk stratification models such as mSMART 3.0, Revised International Staging System (RISS) and others^3^, ^4^, ^5^, ^6^, ^7^ have been developed to define the high‐risk (HR) population based on cytogenetic abnormalities (CAs). Several adverse risk factors that have been established include 1q21 amplification, t(4;14), t(14;16), t(14;20), and 17p deletion,^4^, ^8^ while the prognostic significance of other CAs remains equivocal. The R‐ISS system, in its comprehensive nature, is instrumental in identifying high‐risk features. Its scope extends beyond the integration of cytogenetic abnormalities to include pivotal characteristics such as lactate dehydrogenase (LDH) and Beta‐2 microglobulin levels. These additional parameters bolster the robustness and precision of risk stratification, providing a holistic view of patient prognosis. This multifaceted approach underscores the complexity of multiple myeloma and emphasizes the necessity for a comprehensive risk assessment model akin to the R‐ISS.

*Myc* located on chromosome 8 is recognized as a key oncogene frequently deregulated in various cancers, including diffuse large B‐cell lymphoma, and is associated with poor survival.^9^, ^10^ *Myc* abnormalities, considered secondary CAs, manifesting rearrangement, deletion, amplification, and other complex variations, are able to activate *Myc* expression,^11^ which is a key event in the progression from monoclonal gammopathy of undetermined significance (MGUS) and smoldering myeloma (SMM) to symptomatic myeloma.^12^, ^13^ Myc‐R has been detected in approximately 15% NDMM patients,^14^ suggesting it may adversely affect the outcomes,^15^, ^16^, ^17^, ^18^ while other reports revealed different outcome.^19^, ^20^, ^21^ Therefore, in order to evaluate the prognostic value of different *Myc* abnormalities and their correlation with other high‐risk features, we analyzed the cohort data of MM patients in our center for further understanding and exploration of risk stratification system in MM.

## 2 METHODS AND MATERIALS

### 2.1 Study design and patients

In this retrospective study, NDMM patients were enrolled from May 2009 to September 2022 at Peking Union Medical College Hospital (PUMCH), fulfilling the diagnostic criteria of the 2014 International Myeloma Working Group (IMWG) consensus.^22^ The study flowchart was presented in Figure S1. Clinical data was collected from our myeloma registry database and Electronic Medical Record Analytical Database (EMERALD) in PUMCH.

### 2.2 Fluorescence in situ hybridization (FISH)

Plasma cells were sorted from bone marrow nucleated cells by anti‐CD138 magnetic microbeads (after April 2016). Probes for amp(1q21), t(11;14), t(4;14), t(14;16), and del(17p) were obtained from China Medical Technologies. Although t(14;20) translocation is part of the mSMART classification and is considered a high‐risk feature, it is not included in our routine tests due to its low frequency. The same panels of DNA probes were used for both NDMM and relapsed/refractory multiple myeloma (RRMM). High‐risk cytogenetic abnormalities (HRCAs) were defined by the presence of amp(1q21), t(4;14), t(14;16), or del(17p)(4). A total of 200 interphase cells exhibiting fluorescent signals were examined, and the cut‐off level was set at 10% for chromosome rearrangement and translocation, and 20% for deletion and amplification based on the recommendations from the European Myeloma Network.^23^ However, before the CD138 sort strated, the cutoff values of 1q21 gain, IgH translocation, 17p deletion, and Myc translocation in our center were 5.73%, 4.87%, 3.85%, and 10%, respectively. These values were primarily established through our laboratory's initial investigation and the development of standards. Additionally, Myc aberrations were tested in marrow mononuclear cells (MNCs) using FISH (Vysis Myc Break Apart FISH Probe; Abbott Laboratories, Abbott Park, IL, USA). Myc rearrangement was considered positive when separate FISH signals exceeded 10% (Figure S2). Other abnormalities, such as single‐color probes or increased fusion signals, were classified as deletion or amplification.

### 2.3 Statistical analysis

Categorical variables of baseline characteristics were compared using Fisher's exact test, while the Wilcoxon Rank Sum test was used for continuous variables. The overall response rate (ORR) was defined as a favorable response to front‐line treatment, including stringent complete response (sCR), complete response (CR), very good partial response (VGPR), and partial response (PR), as defined by the international uniform response criteria.^24^ PFS is defined as the time from the start of diagnosis until disease progression or death from any cause, and OS is calculated from the time from diagnosis to death from any causes. Survival curves were plotted using the Kaplan–Meier method and compared between groups using the log‐rank test. Univariate and multivariate Cox hazard regression analysis were developed to identify factors significantly associated with PFS and OS, presenting hazard ratios and 95% confidence intervals. In Cox hazard regression analysis, parameters with *p* value < 0.05 in univariable regression analysis (Table S1) were incorporated in multivariable model. All statistical tests were two‐sided and *p*‐value < 0.05 was considered statistically significant. Statistical analyses were performed using SPSS version 27.0 (SPSS Inc./IBM, Armonk, NY).

## 3 RESULTS

### 3.1 Patient characteristics

The baseline characteristics of 417 NDMM patients were listed in Table 1. Patients with negative *Myc* results were classified as control group. Overall, Myc‐R was detected in 57 (13.7%) patients, while 61 (14.6%) patients presented Myc‐OA, including *Myc* amplification (39, 63.9%), *Myc* deletion (12, 19.7%), and other complex abnormalities (10, 16.4%). Patients with Myc‐R manifested a higher proportion of elevated LDH and extramedullary disease (EMD), indicating an aggressive behavior. Other clinical characteristics between three groups were comparable.

**TABLE 1:** Clinical characteristics of NDMM patients at baseline.

Parameters	Total	Myc‐N	Myc‐R	Myc‐OA	*p*‐Value
*N* (%)	417	299 (71.7%)	57 (13.7%)	61 (14.6%)	
Female/Male	191/226	135/164	27/30	29/32	0.915
Age (years)	62 (54–68)	62 (54–68.0)	59 (54–66)	63 (56–70)	0.254
HGB (g/L)	95 (77–115)	95 (76–115)	92 (79–103)	101 (80–119)	0.406
LDH (U/L)	175 (142.5–222)	175 (144–218)	184 (129–269)	176 (149–204)	0.595
LDH > ULN^a^	66 (15.8%)	43 (14.4%)	16 (28.1%)	7 (11.5%)	0.022
sCr (μmol/L)	84.5 (66–145)	82 (66–143)	93 (71–215)	82 (62.5–130.5)	0.246
Calcium (mmol/L)	2.3 (2.16–2.48)	2.27 (2.16–2.46)	2.36 (2.14–2.52)	2.34 (2.19–2.48)	0.316
Paraproteins					
IgA	104 (24.9%)	70 (23.4%)	15 (26.3%)	19 (31.1%)	0.23
IgG	183 (43.9%)	135 (45.2%)	24 (42.1%)	24 (39.3%)	
IgD	27 (6.5%)	14 (4.7%)	8 (14.0%)	5 (8.2%)	
LC	96 (23.0%)	74 (24.7%)	10 (17.5%)	12 (19.7%)	
Others^b^	7 (1.7%)	6 (2.0%)	0	1 (0.2%)	
ISS					
I	62 (15.3%)	48 (16.4%)	4 (7.1%)	10 (17.2%)	0.458
II	110 (27.1%)	79 (27.1%)	15 (26.8%)	16 (27.6%)	
III	234 (57.6%)	165 (56.5%)	37 (66.1%)	32 (55.2%)	
EMD	103 (24.7%)	68 (22.7%)	22 (38.6%)	13 (21.3%)	0.031
EMD‐B/EMD‐S	66/37	44/24	13/9	9/4	0.863

*Note*: Data are listed as *n* (%) and median (interquartile range, 25%–75%).

Abbreviations: EMD, extramedullary disease; EMD‐B, bone‐related EMD; EMD‐S, soft‐tissue related EMD; HGB, hemoglobin; Ig, Immunoglobulin; ISS, International Staging System; LC, light chain; LDH, lactate dehydrogenase; Myc‐N, no *Myc* abnormalities; Myc‐OA, other *Myc* abnormalities; Myc‐R, *Myc* rearrangement; NDMM, newly diagnosed multiple myeloma; sCr, serum creatinine; ULN, upper limit of normal value.

^a^ ULN = 250 U/L.

^b^ Other Immunoglobulin isotypes include biclonal type and nonsecretory type.

### 3.2 Cytogenetic abnormalities and Myc abnormalities

Among the cohort, 300 patients (71.9%) had been detected with at least one of the following abnormalities: amp(1q21), IgH translocations, del(17p), or *Myc* abnormalities. High‐risk IgH translocations (IgH‐HR) were defined as t(4;14) and/or t(14;16). The occurrence rates of traditional HRCAs, including amp(1q21), del(17p), and IgH‐HR were 42.4% (177 cases), 15.6% (65 cases), and 11.6% (49 cases), respectively. As shown in Table 2, the frequency of concurrent amp(1q21) was lower in the Myc‐N group (36.5%, *p* < 0.001). Moreover, amp(1q21) with ≥4 copies was found in 13 (39.4%) patients with Myc‐OA, which was largely higher than the other groups (*p* = 0.019). Plus, 66.7% patients with Myc‐R had other conventional HRCAs, with amp(1q21) as the most common (61.4%), followed by 17p deletion (15.8%).

**TABLE 2:** Cytogenetics abnormalities and Myc abnormalities.

Parameters	Total	Myc‐N	Myc‐R	Myc‐OA	*p*‐value
*N* (%)	417	299 (71.1%)	57 (13.7%)	61 (14.6%)	
Amp(1q21)	177 (42.4%)	109 (36.5%)	35 (61.4%)	33 (54.1%)	<0.001
3 Copies	141 (79.7%)	93 (85.3%)	28 (80.0%)	20 (60.6%)	0.019
≥4 Copies	36 (20.3%)	16 (14.7%)	7 (19.4%)	13 (39.4%)	
Del(17p)	49 (11.8%)	30 (10.0%)	9 (15.8%)	10 (16.4%)	0.214
IgH translocations					
t(11;14)	64 (15.3%)	43 (14.4%)	10 (17.5%)	11 (18.0%)	0.759
t(4;14)/t(14;16)	65 (15.6%)	49 (16.4%)	6 (9.2%)	10 (16.4%)	

Abbreviations: Amp(1q21), 1q21 amplification; del(17p), 17p deletion; Myc‐N, no *Myc* abnormalities; Myc‐OA, other *Myc* abnormalities; Myc‐R, *Myc* rearrangement.

### 3.3 Treatments and responses

**FIGURE 1:**
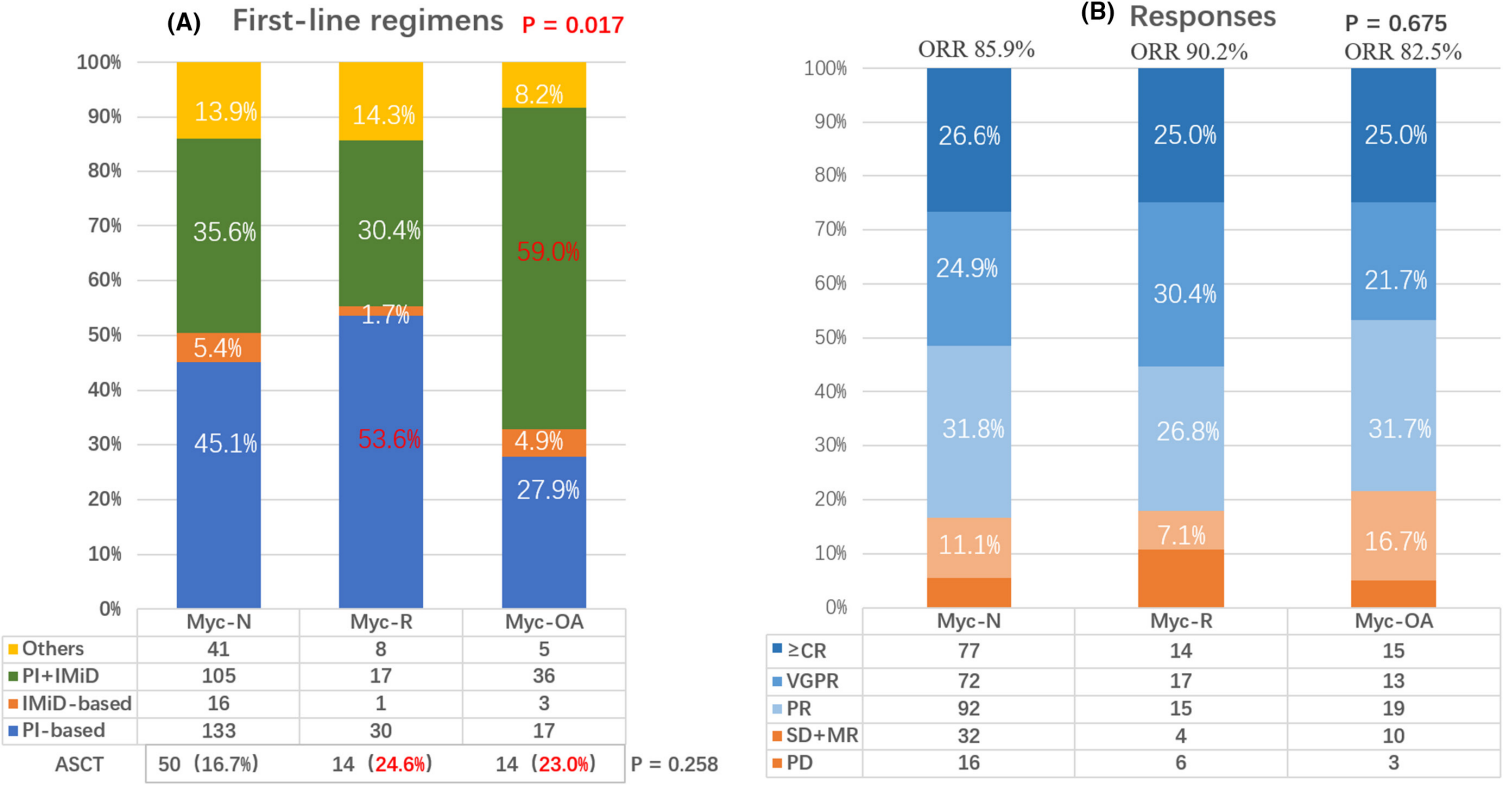
Front‐line regimens and responses. A shows the proportion of four regimens in three groups. PI‐based regimens (blue) were bortezomib‐based or ixazomib‐based; IMiD‐based regimens (orange) were lenalidomide‐based or pomalidomide‐based. The novel regimens include PI and/or IMiD showed no differences in three groups (blue+orange+green). B demonstrates the responses to front‐line treatments in three groups. There are no differences in overall response rate (overall response rate, defined as partial response or better, blue areas).

Treatment response was evaluated in 401 patients (Figure 1). PI‐based regimens consisted of bortezomib or ixazomib, while IMiD‐based regimens included lenalidomide or pomalidomide. The eligible population as recommended to undergo early autologous hematopoietic stem cell transplantation (ASCT). All patients were categorized into three groups: (1) PI/IMiD‐based regimen (either PI or IMiD), (2) PI+IMiD combinations, (3) other treatments. It is evident that 59% patients in Myc‐OA group received PI+IMiD combinations, compared to 30.4% in Myc‐R group and 35.6% in Myc‐N group (*p* = 0.017). However, the proportion of patients receiving novel regimens or ASCT were comparable among three groups (*p* = 0.412, *p* = 0.258). Furthermore, the overall response rate (ORR) of front‐line treatment was 85.9% in Myc‐N group, 90.2% in Myc‐R group, and 82.5% in Myc‐OA group (*p* = 0.675).

### 3.4 Outcomes and survival

**FIGURE 2:**
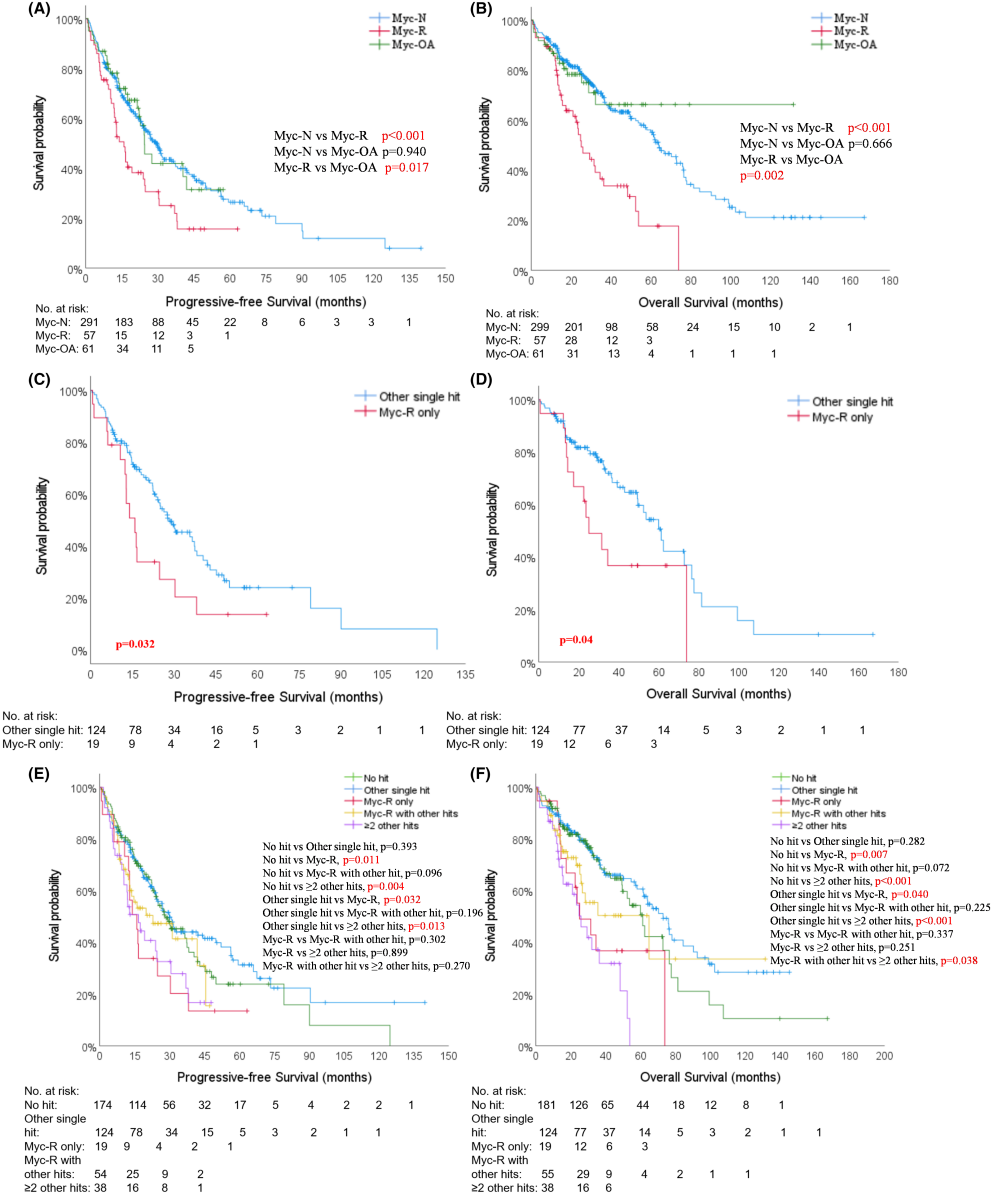
Kaplan–Meier survival analysis of progression‐free survival (PFS) and overall survival (OS). The comparison of PFS and OS among patients with Myc‐N, Myc‐R, and Myc‐OA (A, B); patients with Myc‐R only and other traditional single high‐risk cytogenetic abnormalities (C, D); patients with no hit, traditional single‐hit, Myc‐R only, ≥2 traditional hits, Myc‐R+ traditional hits (E, F).

The median follow‐up time in the entire cohort was 39.7 months (m), with a median PFS of 26.8 m and a median OS of 61.6 m. Only 10 (2.4%) patients were excluded due to missing data (Figure S3). Kaplan–Meier analysis revealed that both PFS and OS were significantly shorter in patients with Myc‐R, compared to that in Myc‐OA and Myc‐N groups (median PFS, 15.9 m vs. 24.5 m vs. 29.8 m, Figure 2A; median OS, 25.1 m vs. unreached vs. 64.1 m, Figure 2B). There were no significant differences between Group Myc‐N and Group Myc‐OA in terms of PFS and OS. Further analysis suggested that patients with Myc‐R had shorter PFS and OS (15.9 m vs. 28.1 m, *p* = 0.032, Figure 2C; 25.1 m vs. 61.2 m, *p* = 0.040, Figure 2D) than those with traditional single‐hit HRCA. Moreover, all patients were classified into five groups: no hit (*n* = 181), traditional single HRCA (*n* = 124), Myc‐R alone (*n* = 19), ≥2 traditional HRCAs (*n* = 55), ≥2 hits including Myc‐R or traditional HRCAs (*n* = 38). It was evident that PFS and OS were shorter in patients with Myc‐R or ≥2 hits compared to patients in other three groups (Figure 2E,F).

Overall, soft‐tissue related EMD (EMD‐S), Myc‐R and HRCA ≥ 2 hits had an adverse impact on PFS and OS. Patients receiving ASCT after front‐line regimens had longer PFS and OS. Additionally, patients in PIs or IMiDs containing groups had longer PFS. Advanced age, elevated LDH and hypercalcemia were identified as risk factors for OS. Therefore, Myc‐R rather than Myc‐OA, ASCT were independent prognostic factors for NDMM patients.

In the layered multivariable Cox hazard regression model (Table S2), where the first‐line treatment regimen was considered as a stratified factor, both traditional chemotherapy and novel regimens based on PIs and/or IMiDs were included. Sensitivity analyses revealed that Myc‐R continued to have a significant impact on both PFS and OS. Specifically, Myc‐R was associated with a hazard ratio (HR) of 1.540 for PFS (95% confidence interval [CI] 1.054–2.249, *p* = 0.026) and an HR of 2.525 for OS (95% CI 1.649–3.866, *p* < 0.001) (Table 3).

**TABLE 3:** Multivariable Cox hazard regression analysis for PFS and OS.

Parameters	PFS			OS		
	HR	95% CI	*p*‐Value	HR	95% CI	*p*‐Value
Age (years)						
<50				0.699	0.431–1.134	0.147
>70				1.608	1.064–2.430	0.024
Calcium (mmol/L)	1.009	0.999–1.019	0.086	1.013	1.004–1.023	0.008
HGB (g/L)	0.800	0.589–1.087	0.153	1.015	0.717–1.436	0.993
HGB < 100 g/L	0.889	0.65–1.215	0.459	1.167	0.810–1.680	0.406
LDH (U/L)	1.397	0.978–1.997	0.066	1.002	1.001–1.003	0.007
LDH > ULN*	1.232	0.852–1.781	0.268	1.425	0.949–2.142	0.088
EMD						
EMD‐B	1.159	0.797–1.685	0.440	1.438	0.904–2.287	0.125
EMD‐S	2.629	1.755–3.938	<0.001	2.222	1.430–3.452	<0.001
ISS stage						
II	1.684	1.013–2.799	0.054	1.838	0.976–3.461	0.059
III	1.589	0.983–2.569	0.059	1.878	1.024–3.444	0.052
Del(17p)	1.452	0.996–2.117	0.052	1.448	0.942–2.227	0.091
*Myc*						
Myc‐R	1.937	1.332–2.815	<0.001	2.907	1.922–4.397	<0.001
Myc‐OA	1.089	0.685–1.729	0.719	1.191	0.683–2.078	0.538
HRCA						
Single‐hit	1.152	0.847–1.569	0.367	1.258	0.872–1.814	0.22
≥2 hits	1.424	1.001–2.027	0.049	2.017	1.329–3.060	<0.001
The first‐line regimens^a^						
Chemotherapy versus novel	0.509	0.367–0.706	<0.001	0.783	0.527–1.163	0.226
ASCT	0.458	0.302–0.693	<0.001	0.486	0.282–0.836	0.009

Abbreviations: 95% CI, 95% confidence interval; ASCT, autologous hematopoietic stem cell transplantation; EMD, extramedullary disease; EMD‐B, bone‐related EMD; EMD‐S, soft‐tissue related EMD; HGB, hemoglobin; HR, hazard ratio; ISS, International Staging System; LDH, lactate dehydrogenase; Myc‐OA, other *Myc* abnormalities; Myc‐R, *Myc* rearrangement; ORR, overall response rate; OS, overall survival; PFS, progressive‐free survival.

^a^ The first‐line regimens include traditional chemotherapy and novel regimen based on proteasome inhibitors (PIs) and/or immunomodulatory drugs (IMiDs).

*ULN=250U/L.

Furthermore, the restricted Kaplan–Meier survival analysis of PFS and OS was performed on 216 patients diagnosed between 2014 and 2020 in this cohort (Figure S4). The patients were divided into three groups: the Myc‐N group with 159 patients (73.6%), the Myc‐R group with 29 patients (13.4%), and the Myc‐OA group with 28 patients (13.0%). Accordingly, the median PFS for the Myc‐N, Myc‐R, and Myc‐OA groups were 29.80, 16.63, and 22.27 months, respectively. The median OS for the Myc‐N group was 64.1 months, for the Myc‐R group it was 34.47 months, while it was not reached for the Myc‐OA group. The median follow‐up times for the Myc‐N, Myc‐R, and Myc‐OA groups were 48.37, 49.43, and 47.27 months respectively.

### 3.5 The new risk stratification models

**FIGURE 3:**
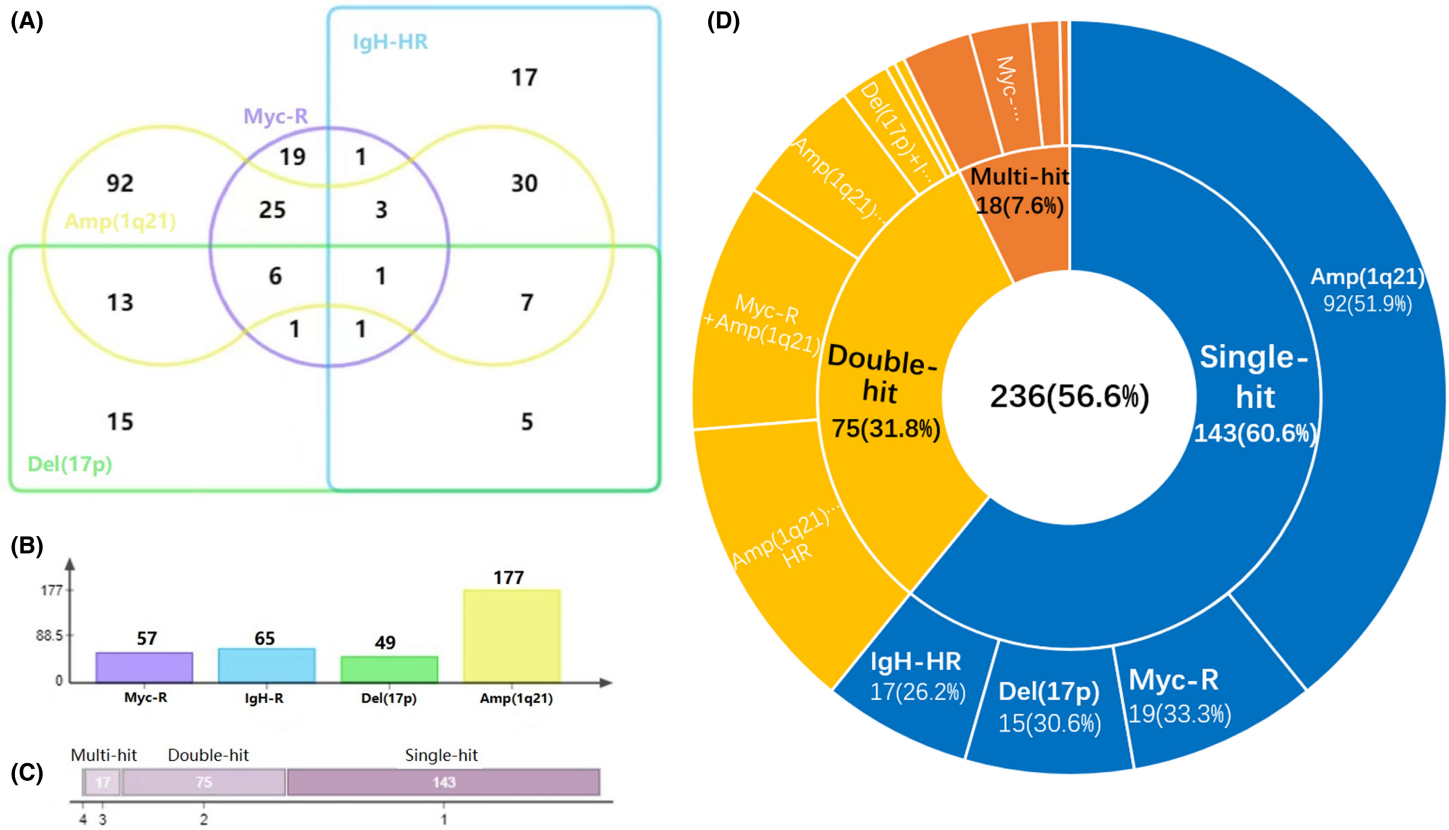
The complex association of Myc‐R with traditional HRCAs. The different overlapping areas represents respective co‐occurrence of Myc‐R and HRCAs (A). When Myc‐R were regarded as a new high‐risk hit, the axis shows the frequencies of four hits in the whole cohort (B). Single‐hit is defined with one of four high‐risk hits only; double‐hit contains two of these hits, and ≥3 hits are regarded as multi‐hit (C). 236 patients (56.6%) with CA and detailed HRCAs are displayed (D). Amp(1q21), 1q21 amplification including 3 copies and ≥4 copies; Del(17p), 17p deletion; HRCA, high‐risk cytogenetic abnormalities; IgH‐HR, high‐risk IgH translocations including t(4;14) and t(14;16); Myc‐R, Myc rearrangement.

**FIGURE 4:**
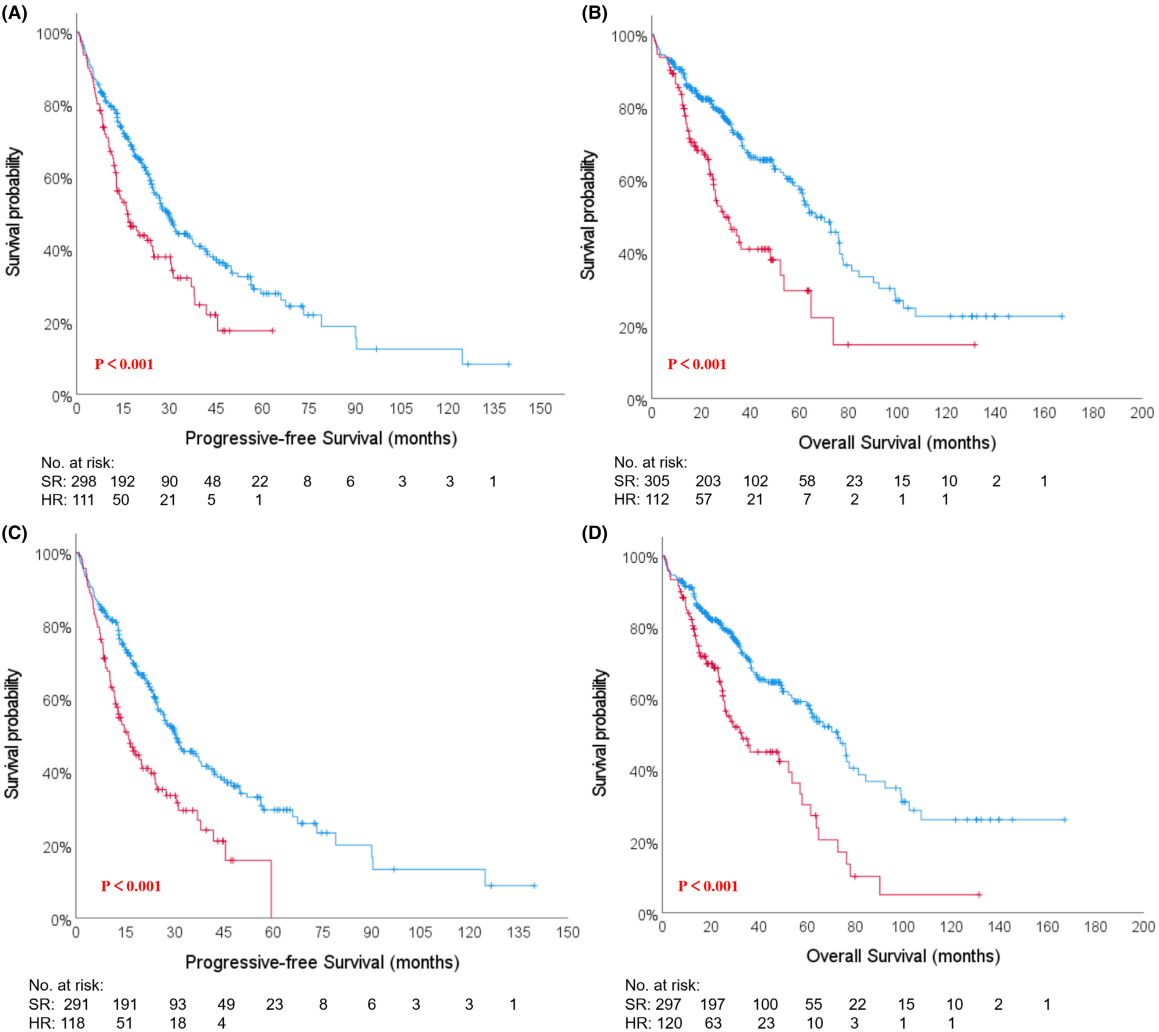
Kaplan–Meier survival analysis of progression‐free survival (PFS) and overall survival (OS) in the new risk stratification models. In the model 1, more than 2 hits was the high‐risk factor. There were 112 (26.9%) patients in high‐risk (HR)group and 305 (73.1%) in standard‐risk (SR) group. Median PFS (A) were 16.4 months versus 29.8 months (*p* < 0.001); median OS (B) were 29.3 months versus 66.7 months (*p* < 0.001). In the model 2, more than two hits and/or EMD‐S were the high‐risk factors. There were 120 (28.8%) patients in high‐risk (HR)group and 297 (71.2%) in standard‐risk (SR) group. Median PFS (C) were 15.9 months versus 30.4 months (*p* < 0.001); median OS (D) were 32.9 months versus 72.9 months (*p* < 0.001).

Based on the previous survival analysis, Myc‐R was identified as a novel HRCA. The intricate relationship between Myc‐R and the other three traditional HRCAs was depicted in Figure 3. Approximately two‐thirds of patients with Myc‐R were found to have other HRCAs (Figure 3A). Notably, Amp(1q21) remained the most prevalent abnormality in this cohort (Figure 3B). Furthermore, the distribution of patients categorized as single‐hit, double‐hit, and multi‐hit was 60.6%, 31.8%, and 7.6%, respectively (Figure 3C,D), following the integration of Myc‐R into the new risk stratification model for NDMM. In the model 1 (Figure 4A,B), Myc‐R or ≥2 HRCAs were identified as high‐risk factors. Therefore, all patients in our cohort were divided into standard‐risk (SR) and high‐risk (HR) groups. HR patients (*n* = 112, 26.9%) did have shorter survival (PFS 16.4 m vs. 29.8 m, *p* = 0.001; OS 29.3 m vs. 66.7 m, *p* < 0.001, Figure 4). As a result, the internal validation results indicated outstanding predictive performance of a new risk stratification model for patients with NDMM. In addition to the impact of HRCAs, the presence of EMD‐S also significantly influences patient prognosis. As such, factors such as the presence of more than two hits and/or EMD‐S were incorporated into the second model as high‐risk indicators (Figure 4C,D). Out of the total patient population, 120 patients (28.8%) were categorized into the HR group, while 297 patients (71.2%) fell into the SR group. The median PFS was significantly different between the two groups, with 15.9 months for the HR group versus 30.4 months for the SR group (*p* < 0.001). Similarly, a significant difference was observed in the median OS, with 32.9 months for the HR group compared to 72.9 months for the SR group (*p* < 0.001).

## 4 DISCUSSION

It is difficult to clearly delineate the high‐risk population of multiple myeloma, although several authoritative consensuses updated the risk stratification criteria from time to time. In a series of prognostic parameters, specific chromosomal abnormalities (CAs) play a key role in predicting clinical outcome. However, the value of single HRCA mainly amp(1q21), del(17p), t(4;14), or t(14;16) was paradoxical because of the heterogeneity of survival in patients with same HRCA. Therefore, it is indeed essential to identify a key factor for high‐risk MM patients. Emphasis should be placed on the various combinations of adverse cytogenetic abnormalities, and efforts should be made to refine our understanding of these combinations. Here we validated the undisputed negative impacts of *Myc* rearrangement in NDMM patients,^17^ who manifested high tumor burden with more ISS III, elevated LDH, soft‐tissue EMD. Indeed, those with Myc‐R demonstrated an aggressive behavior and shorter survival. Yet, *Myc* detection was overlooked. In our study, 13.7% NDMM patients were Myc‐R positive compared to 4%–18% in other reports.^14^ Next‐generation sequencing (NGS) could even identify Myc‐R in 36% MM patients.^25^ Sharma et al.^16^ from Mayo Clinic found a higher detection rate and more subtypes of *Myc* structural variants (SV) using NGS than FISH. To our understanding Myc‐R tested by FISH probes plays significant impact on suruvial.^26^ Patients with Myc‐R had shorter OS compared to those without Myc‐R (5.3 years vs. 8.0 years, *p* < 0.001).^18^ In the UK MRC Myeloma IX trial, median PFS and OS was 11.8 months vs 20.0 months (*p* = 0.016) and 19.7 months vs 55.8 months (*p* = 0.043) in patients with and without *Myc* rearrangement, respectively.^27^

In addition, it is the first time that we clarified Myc‐R rather than other abnormalities was associated with poor prognosis in MM patients. While rearrangement and amplification are the most common in *Myc* abnormalities,^28^, ^29^ patients with *Myc* amplification, included in the Myc‐OA group, had similar baseline characteristics, treatment response rates and outcomes to the Myc‐N population. The potential mechanism of adverse impact of Myc‐R remains largely unknown. One possible mechanism is that Myc‐R brings about the juxtaposition of a super‐enhancer adjacent to *Myc*, resulting in the increased expression of *Myc* mRNA.^30^, ^31^ *Myc* is considered a promising therapeutic target and several novel agents are currently under developed,^32^, ^33^ although the clinical efficacy is yet to be demonstrated (NCT05263583).

Indeed, with advancements in detection technology and the development of novel drugs, the risk stratification systems for MM are evolving, and there is ongoing controversy regarding the definition of high‐risk populations.^4^, ^7^, ^20^, ^34^ It is now recognized that the co‐occurrence of two or more HR factors, known as “double‐hit” and “triple‐hit”, indicates a dismal prognosis.^35^, ^36^ In our cohort, survival in patients with traditional single‐hit abnormalities (124 cases) did not shown difference compared to those without any hit (174 cases) (Figure 2E,F). However, median PFS and OS of patients with Myc‐R alone were much shorter than those with single HRCA (*p* = 0.032, *p* = 0.04 respectively), while similar to those with double‐hit HRCAs (Figure 2E,F, *p* = 0.899; *p* = 0.251). Myc‐R was identified as an independent adverse prognostic factor in both univariable and multivariable Cox hazard regression analysis, consistent with the findings of another study.^27^

In our cohort, two‐thirds of patients with Myc‐R accompanying traditional HRCAs, with the most common being 1q21 amplification (61.4%), followed by 17p deletion (15.8%). Worse clinical outcomes were observed in the subgroup with the concomitant of amp(1q21) and Myc‐R.^17^, ^19^ Our previous study also suggested patients with amp(1q21) and Myc‐R only had a median OS of 9.3 months,^17^ while single amp(1q21) did not negatively affect survival. Therefore, Myc‐R or ≥2 traditional HRCAs were included as HR factors in our new risk stratification model for NDMM, which was internally validated using our cohort (Figure 4A,B).

Furthermore, to account for variations in patients receiving combinations of PIs and IMiDs across the groups, we conducted sensitivity analyses specifically on patients receiving consistent treatments across groups to mitigate a significant confounding factor associated with the outcome (Table S2). Even when considering the first‐line treatment regimen as a stratified factor in the multivariable Cox hazard regression model, which encompassed both traditional chemotherapy and novel regimens based on PIs and/or IMiDs, Myc‐R continued to exert a significant impact on both PFS and OS.

Additionally, in light of the censoring within the cohort, we performed a restricted analysis on patients diagnosed between 2014 and 2020 to ensure the robustness of the results (Figure S4). This supplementary analysis further corroborated our findings. Unfortunately, the validity of the new model remains unverified by external data. The primary objective of this investigation was to evaluate the influence of Myc rearrangement on clinical outcomes in individuals with myeloma. It is crucial to emphasize that this study contributes to the ongoing exploration of high‐risk cytogenetics, rather than providing a definitive conclusion. However, there are other limitations in this study. Firstly, its retrospective nature, a small cohort of patients and relatively short follow‐up time may hinder the generalizability of our results. Secondly, our findings are only applicable to patients with Myc‐R by FISH using the *Myc* break apart probe. The cut‐off value of defining positive Myc‐R was based on our single institution's data. Whether companion chromosomes of *Myc* translocation play different roles needs to be further explored. Thirdly, the new risk model has not yet validated in an independent cohort. Lastly, due to the heterogeneity of treatment regimens and limited sample sizes, this study has yielded inconclusive results regarding whether the adverse impact can be mitigated by novel agents.

## 5 CONCLUSION

In this study, about 10%–15% NDMM patients develop *Myc* rearrangement, a crucial adverse factor worthy of attention. We first demonstrate that only *Myc* rearrangement rather than other abnormalities emerges as an independent factor associated with inferior survival. As novel agents have improved survival of MM patients, single traditional high‐risk cytogenetic abnormalities are not correlated with poor prognosis. Consequently, Myc‐R along with double or multiple HRCAs could be potentially identified as crucial factors in the new risk stratification model for MM patients.

## AUTHOR CONTRIBUTIONS

**Xianghong Jin:** Data curation (lead); formal analysis (lead); writing – original draft (lead). **Hui Li:** Data curation (supporting); investigation (supporting); methodology (equal). **Shuangjiao Liu:** Data curation (supporting); investigation (lead). **Yuhang Song:** Data curation (supporting); investigation (supporting). **Fujing Zhang:** Investigation (supporting); supervision (supporting). **Ziping Li:** Investigation (supporting); methodology (supporting). **Junling Zhuang:** Conceptualization (lead); funding acquisition (lead); resources (lead); writing – review and editing (lead). **Dingding Zhang:** Validation (equal); writing – review and editing (equal).

## FUNDING INFORMATION

This study was funded by the Capital Health Development Scientific Research Fund (Grant No. 2022‐2‐4013), and National High Level Hospital Clinical Research Funding (2022‐PUMCH‐B‐048).

## CONFLICT OF INTEREST STATEMENT

The authors disclosed no potential conflict of interests.

## ETHICS STATEMENT

All procedures performed in studies involving human participants were in accordance with the ethical standards of the Ethic Committee of Peking Union Medical College Hospital and with the 1964 Helsinki declaration. The Ethics Committee granted an exemption for written informed consent. The protocol number is K3855.

## Supporting information


Figure S1

Table S1

## Data Availability

The data that support the findings of this study are available from the corresponding author, Junling Zhuang, upon reasonable request.
