# Supplementary material for: Myc rearrangement redefines the stratification of high‐risk multiple myeloma
Source: Cancer Med. 2024 Jun 7;13(11):e7194. doi: 10.1002/cam4.7194 (PMC11157166; doi:10.1002/cam4.7194)
Supplement: Supplementary file 1 — Figure S1 [file CAM4-13-e7194-s002.docx]

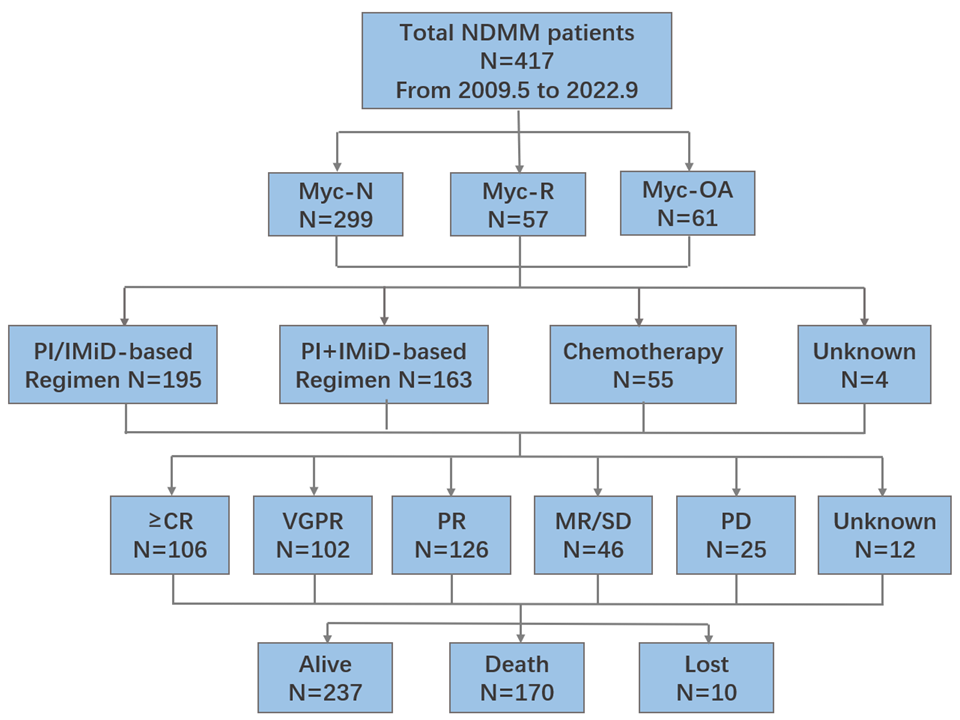


Supplementary figure 1. The study flowchart.

Abbreviations: NDMM, newly diagnosed multiple myeloma; Myc-N, no abnormalities of Myc; Myc-R, Myc rearrangement; Myc-OA, other Myc abnormalities; PI, proteasome inhibitor; IMiD, immunomodulatory drug; CR, complete response; VGPR, very good partial response; PR, partial response; MR, minimal response; SD, stable disease; PD, progressive disease.


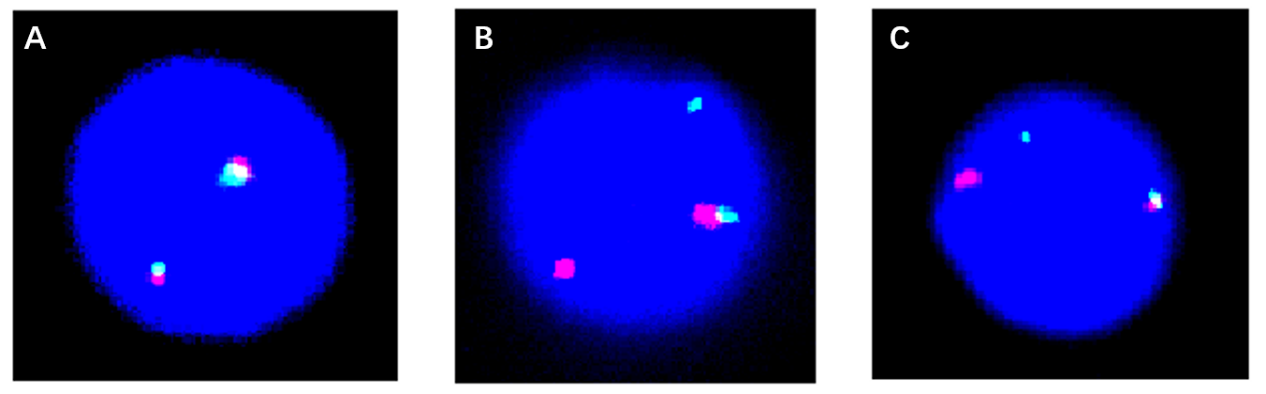


Supplementary figure 2. Representative Myc rearrangement manifestation by FISH. The red and green signals represent two different DNA sequences that have been labeled with red and green fluorescent dyes, respectively. Separate signal refers to the presence of two distinct signals in a cell, each representing a copy of the MYC gene. This is typically seen in normal cells where there are two copies of each gene. If these signals overlap and appear yellow, it indicates that the sequences are located very close together or have fused, which can be indicative of a genetic rearrangement. The negative detection of Myc was observed with two infusion signals (A). The result of Myc rearrangement shows an infusion signal and two small separation signals (B, C).


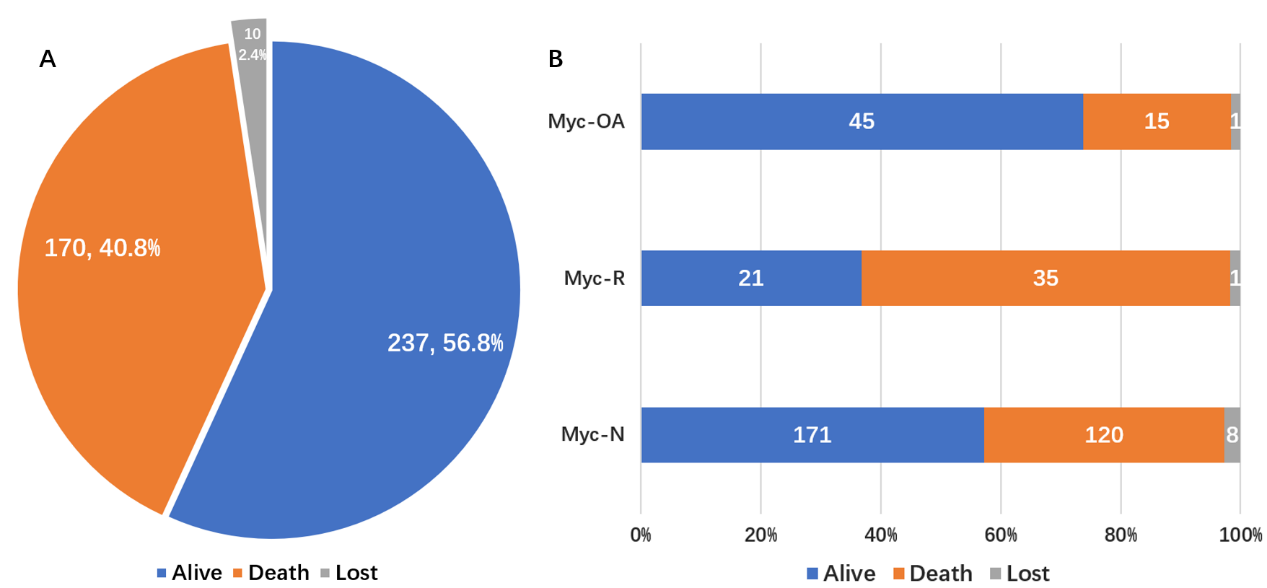


Supplementary figure 3. The outcomes of MM patients. As of the follow-up date, 237 patients (56.8%) are alive in the whole cohort (A). The outcomes of patients in three groups are shown (B).


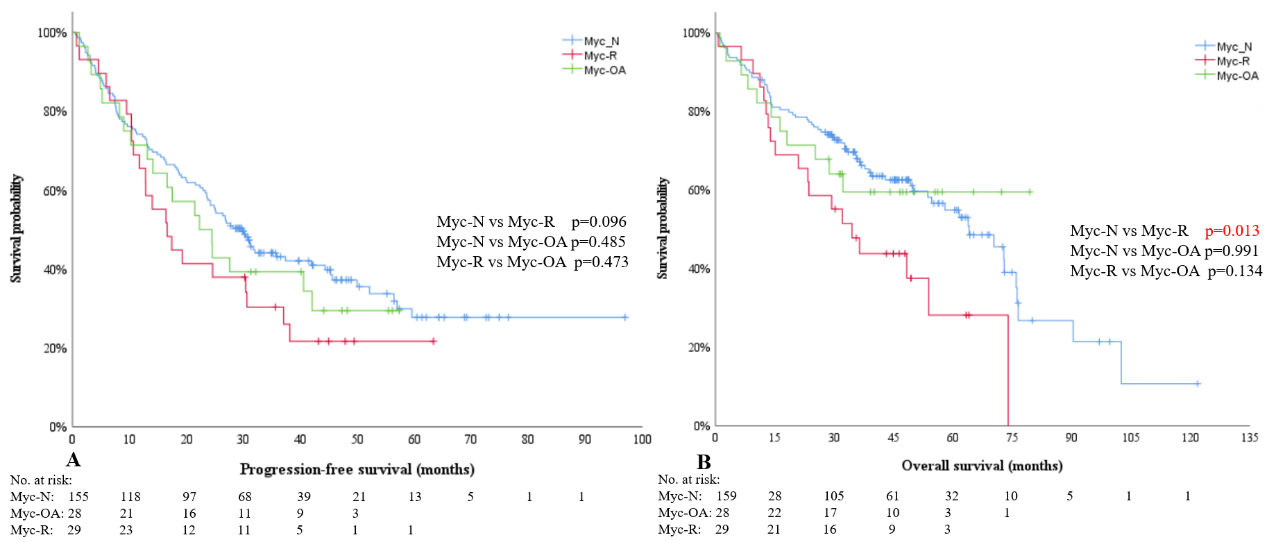


Supplementary figure 4. The restricted Kaplan-Meier survival analysis of PFS and OS on 216 patients diagnosed between 2014 and 2020. There were 159 (73.6%) patients in Myc-N group, and 29 (13.4%) patients in Myc-R group, and 28 (13.0%) patients in Myc-OA group. Median PFS were 29.80 months vs 16.63 months vs 22.27 months; median OS were 64.1 months vs 34.47 vs not reached; median follow-up time were 48.37 months vs 49.43 months vs 47.27 months
