# Supplementary material for: Myc rearrangement redefines the stratification of high‐risk multiple myeloma
Source: Cancer Med. 2024 Jun 7;13(11):e7194. doi: 10.1002/cam4.7194 (PMC11157166; doi:10.1002/cam4.7194)
Supplement: Supplementary file 2 — Table S1 [file CAM4-13-e7194-s001.docx]

Supplementary Table 1. Univariable Cox hazard regression analysis for PFS and OS.

| Parameters | PFS | | | OS | | |
| --- | --- | --- | --- | --- | --- | --- |
|  | HR | 95%CI | p-value | HR | 95%CI | p-value |
| Sex (female vs. male) | 1.022 | 0.789-1.323 | 0.869 | 1.057 | 0.778-1.436 | 0.723 |
| Age (years) | 1.010 | 0.997-1.023 | 0.145 | 1.024 | 1.007-1.042 | 0.007 |
| >70 | 1.13 | 0.798-1.599 | 0.491 | 1.545 | 1.051-2.27 | 0.027 |
| <50 | 0.796 | 0.549-1.154 | 0.229 | 0.633 | 0.397-1.01 | 0.055 |
| Calcium (mmol/L) | 1.010 | 1.001-1.020 | 0.037 | 1.012 | 1.003-1.022 | 0.011 |
| sCr (μmol/L) | 1.0 | 1.001-1.001 | 0.351 | 1.001 | 1.0-1.001 | 0.073 |
| HGB (g/L) | 0.989 | 0.983-0.994 | ＜0.001 | 0.992 | 0.986-0.999 | 0.025 |
| HGB<100g/L | 1.449 | 1.11-1.893 | 0.006 | 1.182 | 0.867-1.611 | 0.291 |
| LDH (U/L) | 1.002 | 1.001-1.003 | 0.001 | 1.003 | 1.002-1.005 | ＜0.001 |
| LDH>ULN^&^ | 1.438 | 1.026-2.016 | 0.035 | 2.148 | 1.494-3.089 | ＜0.001 |
| EMD |  |  |  |  |  |  |
| EMD-B | 1.151 | 0.811-1.633 | 0.432 | 1.320 | 0.861-2.022 | 0.203 |
| EMD-S | 2.932 | 1.996-4.306 | ＜0.001 | 2.580 | 1.713-3.886 | ＜0.001 |
| Paraproteins |  |  |  |  |  |  |
| IgG | 0.894 | 0.647-1.236 | 0.498 | 0.878 | 0.603-1.279 | 0.499 |
| LC | 0.770 | 0.526-1.126 | 0.177 | 0.804 | 0.519-1.247 | 0.330 |
| IgD | 1.299 | 0.773-2.182 | 0.323 | 0.940 | 0.496-1.780 | 0.849 |
| Others | 1.104 | 0.443-2.755 | 0.831 | 1.325 | 0.475-3.697 | 0.591 |
| ISS |  |  |  |  |  |  |
| II | 2.203 | 1.259-3.250 | 0.004 | 2.257 | 1.154-4.416 | 0.017 |
| III | 2.211 | 1.436-3.406 | ＜0.001 | 2.365 | 1.265-4.422 | 0.007 |
| Amp(1q21) | 1.260 | 0.973-1.631 | 0.08 | 1.356 | 0.997-1.844 | 0.052 |
| 3 Copies vs 4 Copies | 0.984 | 0.609-1.591 | 0.948 | 0.815 | 0.460-1.444 | 0.483 |
| Del(17p) | 1.725 | 1.203-2.475 | 0.003 | 1.650 | 1.090-2.496 | 0.018 |
| IgH-HR^#^ | 0.881 | 0.592-1.311 | 0.532 | 1.531 | 0.88-2.665 | 0.132 |
| *Myc* |  |  |  |  |  |  |
| Myc-R | 1.780 | 1.261-2.513 | 0.001 | 2.630 | 1.729-3.999 | ＜0.001 |
| Myc-OA | 0.979 | 0.651-1.474 | 0.920 | 1.300 | 0.711-2.376 | 0.395 |
| HRCA |  |  |  |  |  |  |
| Single hit | 1.238 | 0.923-1.660 | 0.154 | 1.347 | 0.945-1.922 | 0.100 |
| ≥2 hits | 1.667 | 1.188-2.340 | 0.003 | 2.238 | 1.516-3.304 | ＜0.001 |
| The front-line regimen^*^ |  |  |  |  |  |  |
| Chemotherapy vs novel | 0.532 | 0.391-0.726 | ＜0.001 | 0.802 | 0.554-1.160 | 0.241 |
| ASCT | 0.425 | 0.287-0.632 | ＜0.001 | 0.420 | 0.251-0.703 | ＜0.001 |

^&^ULN= 250U/L

^#^IgH-HR: high risk IGH translocations include t(4;14) and t(14;16).

^*^The first-line regimens include traditional chemotherapy and novel regimen based on proteasome inhibitors (PIs) and/or immunomodulatory drugs (IMiDs).

Abbreviations: PFS, progressive-free survival; OS, overall survival; HR, hazard ratio；95% CI, 95% confidence interval; sCr, serum creatinine; HGB, hemoglobin; LDH, lactate dehydrogenase; ULN, upper limit of the normal value; EMD, extramedullary disease; EMD-B, bone-related EMD; EMD-S, soft tissue-related EMD; Ig, Immunoglobulin; ISS, International Staging System; 1q21 G/A, 1q21 gain or amplification; Del(17p), 17p deletion; Myc-R, *Myc* rearrangement; Myc-OA, other Myc abnormalities; HRCA, high-risk cytogenetic abnormality; ASCT, autologous hematopoietic stem cell transplantation.

Supplementary Table 2. Layered multivariable Cox hazard regression analysis for PFS and OS.

Layered factor: the first-line regimens include traditional chemotherapy and novel regimen based on proteasome inhibitors (PIs) and/or immunomodulatory drugs (IMiDs).

| Parameters | PFS | | | OS | | |
| --- | --- | --- | --- | --- | --- | --- |
|  | **HR** | **95%CI** | **p-value** | **HR** | **95%CI** | **p-value** |
| Age (years) | 1.011 | 0.997-1.026 | 0.120 | 1.029 | 1.011-1.048 | 0.001 |
| Calcium (mmol/L) | 1.008 | 0.998-1.018 | 0.116 | 1.012 | 1.001-1.022 | 0.029 |
| HGB (g/L) | 1.001 | 0.995-0.988 | 0.120 | 0.997 | 0.989-1.005 | 0.413 |
| LDH (U/L) | 1.002 | 1.000-1.003 | 0.026 | 1.002 | 1.001-1.004 | 0.004 |
| EMD |  |  |  |  |  |  |
| EMD-B | 1.172 | 0.796-1.726 | 0.421 | 1.342 | 0.823-2.187 | 0.239 |
| EMD-S | 2.836 | 1.881-4.277 | ＜0.001 | 2.369 | 1.512-3.710 | ＜0.001 |
| ISS stage |  |  |  |  |  |  |
| II | 1.681 | 0.994-2.844 | 0.053 | 1.824 | 0.946-3.515 | 0.073 |
| III | 1.526 | 0.913-2.550 | 0.107 | 1.686 | 0.878-3.241 | 0.117 |
| Del(17p) | 1.420 | 0.969-2.082 | 0.072 | 1.435 | 0.919-2.241 | 0.112 |
| *Myc* |  |  |  |  |  |  |
| Myc-R | 1.540 | 1.054-2.249 | 0.026 | 2.525 | 1.649-3.866 | ＜0.001 |
| Myc-OA | 1.244 | 0.793-1.952 | 0.342 | 1.307 | 0.727-2.349 | 0.372 |

Abbreviations: PFS, progressive-free survival; OS, overall survival; HR, hazard ratio；95% CI, 95% confidence interval; Myc-R, *Myc* rearrangement; Myc-OA, other *Myc* abnormalities; HGB, hemoglobin; LDH, lactate dehydrogenase; ISS, International Staging System; EMD, extramedullary disease; EMD-B, bone-related EMD; EMD-S, soft tissue-related EMD; ASCT, autologous hematopoietic stem cell transplantation.
